# An update on paediatric traumatic brain injury in the emergency department: a narrative review

**DOI:** 10.1007/s00431-025-06345-9

**Published:** 2025-08-02

**Authors:** Sara Alsuwais, Charlotte Kennedy, Silvia Bressan, Mark Lyttle, Richard Body

**Affiliations:** 1 Department of Emergency Medical Services, College of Applied Medical Sciences, King Saud Bin Abdulaziz University for Health Sciences, Riyadh, Saudi Arabia; 2 Division of Cardiovascular Sciences, Faculty of Biology, Medicine and Health, University of Manchester, Manchester, UK; 3 Health Education North West, Manchester, UK; 4 University of Padova, Padua, Italy; 5 Emergency Department, Bristol Royal Hospital for Children, Bristol, UK; 6 Research in Emergency Care Avon Collaborative Hub (REACH), University of the West of England, Bristol, UK; 7 Manchester Royal Infirmary, Manchester, UK; 8 King Abdullah International Medical Research Center (KAIMRC), Riyadh, Saudi Arabia

**Keywords:** Paediatric traumatic brain injury, Emergency department, Clinical decision rules

## Abstract

Traumatic brain injury (TBI) affects an estimated 330,000 to 500,000 children annually in England and Wales and represents a significant burden on healthcare systems. Presentations range from mild to severe, and each case presents unique challenges to the Emergency Department (ED) clinicians. Most paediatric TBIs are mild, and several validated clinical decision rules (CDRs) such as PECARN help guide CT use. Whilst these rules show good sensitivity, clinician gestalt appears to have better specificity. Not all children with mild TBI require neuroimaging, yet many experience persistent symptoms that require clear discharge advice and follow-up planning. Emerging tools such as fast MRI and blood-based biomarkers may enhance early diagnosis and reduce unnecessary CT use, but these remain investigational. Importantly, mild TBI is increasingly recognised as a condition that may lead to prolonged recovery in a significant proportion of children, highlighting the need for tailored counselling and follow-up. In contrast, the ED approach to moderate and severe TBI prioritises early stabilisation and prevention of secondary injury, though a full review of critical care management is beyond the scope of this paper.

This review summarises current evidence relevant to ED-based assessment, imaging decisions, and early management of paediatric TBI, with a focus on mild presentations. We also highlight areas of emerging evidence and identify priority research gaps, including validation of prediction tools in preverbal children, care of neurodivergent populations, and real-world implementation of advanced diagnostics. Finally, we acknowledge the variability in access to imaging and decision-support tools across healthcare settings, and the need for context-specific strategies that support equitable care.

**Table:** 

**What is Known:** • *Most paediatric TBIs are mild, and clinical decision rules like PECARN support imaging decisions in the ED.* • *Mild TBI can still result in prolonged symptoms requiring tailored discharge advice and follow-up.*
**What is New:** • *Emerging tools such as fast MRI and blood-based biomarkers may improve diagnosis but remain investigational.* • *Priority gaps include care strategies for neurodivergent children and preverbal populations in real-world ED settings.*

**What is Known:**

• *Most paediatric TBIs are mild, and clinical decision rules like PECARN support imaging decisions in the ED.*

• *Mild TBI can still result in prolonged symptoms requiring tailored discharge advice and follow-up.*

**What is New:**

• *Emerging tools such as fast MRI and blood-based biomarkers may improve diagnosis but remain investigational.*

• *Priority gaps include care strategies for neurodivergent children and preverbal populations in real-world ED settings.*

## Introduction

Paediatric traumatic brain injury (TBI) is a common reason for emergency department (ED) attendance and represents a significant source of morbidity, healthcare utilisation, and long-term functional impact. Presentations range from mild, self-limiting injuries to severe TBI requiring urgent stabilisation and specialist care. For ED clinicians, the initial evaluation and decision-making process must balance early identification of clinically important TBI against the risks of over-investigation and unnecessary imaging.

This review focuses primarily on the early recognition, assessment, and management of paediatric TBI in the emergency department. Whilst the emphasis is on mild TBI, selected considerations for severe injury and post-discharge care are briefly included where relevant to ED practice. Key diagnostic strategies, emerging tools, and areas of ongoing uncertainty are highlighted, along with clinical implications and directions for future research.

## Definitions of traumatic brain injury and concussion

The World Health Organization (WHO) Steering Committee on Neurotrauma Prevention and Management defines Traumatic Brain Injury (TBI) as ‘an occurrence of injury to the head arising from blunt or penetrating trauma or from acceleration-deceleration forces’ with one or more symptoms of altered consciousness, amnesia, neuropsychological change, neurological abnormalities on examination or the diagnosis of a skull fracture or intracranial lesion [1].

The term ‘concussion’ is used when there is no evidence or suspicion of structural intracranial injury. In practice, it usually refers to symptoms or altered functional state after a head injury in the absence of abnormalities on standard computed tomography (CT), or when CT is not performed due to low clinical suspicion for structural injury, as most children diagnosed with concussion do not undergo CT scanning [2]. Many authors use the terms ‘mild TBI’ and ‘concussion’ interchangeably, whilst others consider concussion a subset of mild TBI [2]. For clarity, this review follows the terminology used in the original referenced studies. Where source terminology is inconsistent, the term mild TBI is used.

## Classification of TBI severity

There are multiple definitions for TBI severity. In general, in the ED setting, severe TBI is usually defined as a Glasgow Coma Scale (GCS) level of ≤ 8/15, and moderate TBI as a GCS of 9–12/15. The Centers for Disease Control and Prevention (CDC) include a GCS of 13–15 in their definition of mild TBI [3]. The paediatric GCS version is used for preverbal children (Table 1).

**Table 1:** Adult and paediatric Glasgow Coma Scale (GCS)

Adult Glasgow Coma Scale	Score	Paediatric Glasgow Coma Scale	Score
**Eye opening**			
Spontaneous	4	Spontaneous	4
To sound	3	To sound	3
To pain	2	To pain	2
None	1	None	1
**Best verbal response**			
Oriented	5	Age-appropriate vocalisation	5
Confused	4	Cries, irritable	4
Inappropriate words	3	Cries to pain	3
Incomprehensible sounds	2	Moans to pain	2
None	1	None	1
**Best motor response**			
Obeys commands	6	Spontaneous movements	6
Localising pain	5	Withdraws to touch	5
Withdrawal from pain	4	Withdraws to pain	4
Abnormal flexion to pain	3	Abnormal flexion to pain	3
Abnormal extension to pain	2	Abnormal extension to pain	2
None	1	None	1

## Epidemiology of TBI in children

Most global reports estimate an annual incidence of TBI between 47 and 280 per 100,000 population [4]. Mild TBI accounts for the majority of TBI presentations to EDs, with moderate to severe TBI representing only 3%–7% of all cases [4]. Whilst the vast majority of head injuries do not result in hospital admission, and many individuals with head injuries do not seek hospital care, the cost of traumatic brain injury in the United Kingdom (UK) is estimated at 15 billion pounds sterling per year, representing 0.8% of the country’s Gross Domestic Product [5].

## Demographics and mechanisms of TBI

TBI has a bimodal distribution by age, peaking at 0–3 years and 15–18 years [4]. The incidence is evenly distributed between biological sexes until the age of three, when males start to incur more TBI than females at an estimated ratio of 1.8:1 [4]. Mechanisms of injury vary depending on the country, local geography (rural versus city), and child age, but motor vehicle collisions and falls generally account for most cases [4, 6]. Motor vehicle accidents predominantly affect older children and adolescents [6], whilst falls predominate in younger children, particularly those aged 0–5 years [4, 6]. In this younger age group, road traffic accidents account for around half of all fatalities, despite only being responsible for a small proportion of injuries overall [6, 7]. Children from lower socioeconomic backgrounds or those with a history of sustaining injuries are at higher risk of TBI [7]. Non-accidental injuries (NAI), including those caused by physical abuse or neglect, are particularly significant in this context, with higher incidences reported in populations from lower socioeconomic backgrounds [8]. It is important to note that much of the available data on TBI mechanisms comes from studies conducted in hospital or ED settings. As a result, these datasets may underrepresent causes that do not routinely present for acute medical care, such as sport-related injuries, which are a common cause of concussion and mild TBI in children, especially in school-aged and adolescent populations.

## Approach to mild TBI: deciding on neuroimaging

Numerous clinical decision rules (CDRs) have been developed to guide the judicious use of CT. The literature on the subject is vast, but the highest quality CDRs to date are the Canadian Assessment of Tomography for Childhood Head injury (CATCH) (Table 2), the Children’s Head injury ALgorithm for the prediction of Important Clinical Events (CHALICE) (Table 3), and the Pediatric Emergency Care Applied Research Network (PECARN) decision rules (Table 4) [9]. The diagnostic accuracy of each CDR in the original studies and on direct comparison is summarised in Table 5 [10]. A recent multicentre study evaluated the PECARN rule in infants younger than 3 months and found it had high sensitivity and negative predictive value for ciTBI [11].

**Table 2:** Canadian Assessment of Tomography for Childhood Head (CATCH) injury

CT of the head is required only for children with minor head injury* and any one of the following findings:
**High risk (need for neurologic intervention)**
• GCS < 15 at 2 h after injury
• Suspected open or depressed skull fracture
• History of worsening headache
• Irritability on examination
**Medium risk (brain injury on CT scan)**
• **Any sign of basal skull fracture (e.g. hemotympanum, ‘raccoon’ eyes, otorrhoea or rhinorrhoea of the cerebrospinal fluid, Battle’s sign)**
• Large, boggy haematoma of the scalp
• Dangerous mechanism of injury (e.g. motor vehicle crash, fall from elevation ≥ 3 ft [≥ 91 cm] or five stairs, fall from bicycle with no helmet)

*Minor head injury is defined as injury within the last 24 h that is associated with a loss of consciousness, definite amnesia, disorientation, persistent vomiting (more than one episode), or persistent irritability in a child under 2 years of age with a GCS of 13–15

**Table 3:** Children’s Head injury ALgorithm for the prediction of Important Clinical Events (CHALICE)

A computed tomography scan is required if any of the following criteria are present:
**History**
• Witnessed loss of consciousness of > 5 min duration • History of amnesia of > 5 min duration • Abnormal drowsiness (in excess) • ≥ 3 vomits after head injury • Suspicion of non-accidental injury • Seizure after head injury in a patient who has no history of epilepsy
**Examination**
• Glasgow Coma Score < 14 or GCS < 15 if < 1 year old • Suspicion of penetrating or depressed skull injury or tense fontanelle • Signs of a basal skull fracture • Positive focal neurology (defined as any focal neurology, including motor, sensory, coordination or reflex abnormality) • Presence of bruise, swelling or laceration > 5 cm if < 1 year old
**Mechanism**
• High-speed road traffic accident either as pedestrian, cyclist or occupant (defined as accident with speed > 40 m/h) • Fall of > 3 m in height • High-speed injury from a projectile or object
If none of the above variables are present, the patient is at low risk of intracranial pathology

**Table 4:** Paediatric Emergency Care Applied Research Network (PECARN)

Children under 2 years of age	Children 2 years and older
**High-risk group: head CT scan recommended if the following features are present:**	
• Paediatric GCS < 14	• GCS < 14
• Signs of altered mental status	• Signs of altered mental status
• Palpable skull fracture	• Signs of basilar skull fracture
**Moderate-risk group—if none of the above is present, head CT scan or a period of observation is recommended, if any of the following features are present:**	
• Nonfrontal scalp haematoma	• Loss of consciousness
• Loss of consciousness > 5 s	• History of vomiting
• Severe mechanism of injury	• Severe mechanism of injury
• Not acting normally per parents	• Severe headache
**If none of the above are present, a head CT scan is not recommended**

**Table 5:** Comparison of the sensitivity, specificity and number of cases missed for the CATCH, CHALICE, and PECARN clinical decision rules both in their original populations and a homogenous population from New Zealand and Australia

Clinical decision rule	Original derivation study				Babl et al. comparative analysis [10]				
	Sensitivity	Specificity	Number of cases missed	Number of missed cases requiring neurosurgery	Percentage of patients meeting the original derivation criteria	Sensitivity	Specificity	Number of cases missed	Number of missed cases requiring neurosurgery
CATCH*	98.1% (94.6–99.4)	50.1% (48.5–51.7)	3 (*n* = 3866)	0	25%	91.9% (86.5–95.6)	70.4% (69.7–71.0)	13 (*n* = 18,913)	1
CHALICE	98.6% (96.4–99.6)	86.9% (86.5–87.4)	4 (*n* = 22,772)	3	99%	92.5% (87.3–96.1)	78.6% (78.0–79.2)	12 (– = 18,913)	2
PECARN^*¥*^ < 2 years	100.0% (86.3–100.0)	53.7% (51.6–55.8)	0 (*n* = 2216)	0	75%	100.0% (91.6–100.0)	59.1% (57.7–60.5)	0 (*n* = 5046)	0
PECARN ≥ 2 years	96.8% (89.0–99.6)	59.8% (58.6–61.0)	2 (*n* = 6411)	0	76%	99.2% (95.4–100.0)	52.0% (51.1–52.8%)	1 (*n* = 13,867)	0

*Using all 7 (high and medium risk) criteria

¥Validation cohort results from the original study

Concerns have been raised that the use of CDRs might increase neuroimaging rates, particularly in areas with low baseline rates. However, imaging rates did not significantly change at a tertiary hospital in Italy after the adoption of an adapted PECARN rule, and similar work by the PECARN group in the USA showed small decreases in CT rates following the adoption of the rules across multiple sites [12, 13]. A recent systematic review supports the utility of these decision rules, demonstrating that structured implementation strategies can reduce imaging rates without compromising patient safety [14]. However, other studies have shown that head CT rates did not significantly decrease across US hospitals over time, suggesting that decision rule impact may be limited by institutional and cultural factors [15]. Whilst decision rules provide a high-sensitivity, protocol-based approach to avoid missing clinically important TBI, some studies suggest that experienced clinicians may achieve higher specificity through clinical judgement ‘gestalt’. However, the role of gestalt remains controversial, especially in medico-legal contexts where adherence to validated rules may offer more defensible practice. The most pragmatic approach may be a combined one: using decision rules as a safety net, whilst applying clinical judgement in borderline cases or complex presentations. This balance is especially relevant in European practice, where guideline availability may vary [16].

Importantly, none of these decision rules apply when NAI is suspected. In such cases, imaging is often performed not solely to assess for clinically important TBI but also for forensic and safeguarding purposes. The threshold for imaging in suspected NAI should remain low, and the use of skeletal surveys and MRI may be indicated as part of a broader evaluation. Ultimately, the strength of these rules lies in identifying children at very low risk of clinically important TBI, allowing clinicians to avoid unnecessary imaging whilst maintaining diagnostic safety.

## Observation duration for patients without neuroimaging in mild TBI

The incidence of late intracranial haemorrhage is very low among children who are observed, with one study finding that only 1 of 676 patients observed for 4 h had an abnormality on neuroimaging, which did not require neurosurgery [17]. However, observation may reduce CT utilisation for patients who are at intermediate risk of TBI [17, 18]. Guidance from Italy and the UK takes this approach [19, 20]. The National Institute for Health and Care Excellence (NICE) in their 2023 update recommends 4–6 h observation for paediatric patients with head injury who experience one of the specific symptoms included in Table 6 [20]. Similarly, research from the Paediatric Research in Emergency Departments International Collaborative (PREDICT) network has supported the use of observation periods as a safe and effective alternative to imaging in low-risk paediatric patients with head injury, helping to reduce unnecessary radiation exposure whilst ensuring safe management [21]. Professionals should note that families must be aware and informed about the risk of CT scan radiation to children and be updated about the recent recommendations on observation; additionally, they should be involved in the decision-making process [3].

**Table 6:** [20] guidance: indications for immediate CT head scan in paediatric patients under 16 with multiple risk factors following head injury [20]

	• Loss of consciousness lasting more than 5 min (witnessed) • Abnormal drowsiness • 3 or more discrete episodes of vomiting • Dangerous mechanism of injury (high-speed road traffic accident as a pedestrian, cyclist or vehicle occupant, fall from a height of more than 3 m, high-speed injury from a projectile or other object) • Amnesia (anterograde or retrograde) lasting more than 5 min (it will not be possible to assess amnesia in children who are preverbal and is unlikely to be possible in children under 5) • Any current bleeding or clotting disorder
**If, during observation, any of the following risk factors are identified, do a CT head scan within 1 h:**	
	• GCS score of < 15 • Further vomiting • Further episode of abnormal drowsiness
**If none of these risk factors occur during observation, use clinical judgement to determine whether a longer period of observation is needed**	

## Discharging children with normal neuroimaging after mild TBI

In an analysis of 13,543 children admitted with blunt head trauma who had normal initial neuroimaging, the incidence of subsequent neuroimaging abnormalities was 0.12% in children with a GCS of 15 and 0.6% in children with GCS 14, with no children requiring neurosurgery [22]. This suggests that most patients do not require additional observation following normal initial neuroimaging, although it is worth noting that the study excluded patients with coagulopathies and ventricular shunts [22]. Current guidelines from Canada suggest that children with a GCS of 14–15/15 and a normal CT scan can be considered for discharge [23]. European guidelines state that patients with a normal CT scan may be discharged if they have a normal neurological examination and/or have a GCS of 15 [19]. [20] guideline update adds further criteria, including the presence of appropriate social circumstances and the absence of safeguarding concerns. Specifically, the guideline highlights the need to consider the possibility of maltreatment and to ensure that discharge into the home environment is safe and appropriate [20].

In addition to imaging and neurological findings, symptom burden should be factored into discharge decisions. Children with ongoing symptoms such as persistent headache, vomiting, dizziness, or behavioural changes may require further observation or short-stay admission, even if CT findings are normal. Some of these symptoms may be difficult to manage safely in the outpatient setting, and early follow-up may not be immediately available. Discharge should be based not only on scan results but also on the child’s overall clinical status and the capacity of the home setting to support recovery.

## Discharge advice for families of children with mild TBI

A systematic review found that most children with mild TBI fully recover within 2 weeks of injury, but some have a prolonged recovery and experience symptoms beyond 1 month [24]. Parents should be informed about the probability of prolonged full recovery for children with mild TBI, as it may take up to three months [3]. Although many guidelines recommend a period of cognitive and physical rest [25], there is conflicting evidence as to its benefit and optimal duration [24]. The UK Concussion Guidelines for Grassroots Sport recommend that children should return to full activity only when they are symptom-free [26]. However, one trial demonstrated that 5 days of strict rest increased the severity and duration of symptoms with no improvement in functional outcome [27]. There is no evidence to support extended or strict rest. Instead, early, light physical activity has shown benefit: a randomised controlled trial demonstrated that subthreshold aerobic exercise, introduced early, reduced symptom burden, and improved outcomes compared to rest alone [28]. The UK Concussion Guidelines for Grassroots Sport recommend 24–48 h of initial rest, followed by a stepwise symptom-limited return to activity [26]. This graded approach helps children stay below their symptom exacerbation threshold whilst gradually increasing physical and cognitive activity. Full return to normal activity is advised only when the child is symptom-free [26].

Adolescents often take longer to recover from mild traumatic brain injuries due to a higher symptom burden. Most children and adolescents return to school within 10 days without additional support, but severe initial symptoms or reduced activity levels can delay this process. Tailored academic accommodations, such as reduced screen time, frequent breaks, shortened school days, and adjusted workloads, may help [24, 29]. These strategies should be individualised, taking into account the child’s symptom profile, school context, and recovery trajectory. Predictive tools such as the 5Ps clinical risk score can be used to identify children at increased risk of prolonged recovery. This score incorporates five predictors: post-injury headache, sensitivity to noise, fatigue, answering questions slowly, and balance problems. Early identification of higher-risk patients may guide follow-up planning and earlier intervention [30]. A return to activity plan should be thoughtfully developed by the healthcare team, in collaboration with the school team and the child’s family, taking into account each individual’s unique experience. Parents should be made aware of any factors that may prolong their child’s symptoms, including previous TBI, stressors, cognitive impairment, or psychiatric or neurologic disorders [3]. The child brain injury trust also emphasises the importance of sleep quality in patients with head injury and encourages health care providers to educate parents about the role of adequate sleep and sleep hygiene in improving a child’s condition [31].

## Role of additional tools in decision-making for mild TBI

Several emerging modalities have been explored to support decision-making in paediatric mild TBI, including fast magnetic resonance imaging (MRI), point-of-care ultrasound (POCUS), near-infrared spectroscopy (NIRS), and blood-based biomarkers [32]. Fast brain MRI, already used for hydrocephalus monitoring and other neurological assessments, has shown increasing potential in evaluating head trauma. MRI avoids ionising radiation and offers improved sensitivity to subtle lesions such as microhaemorrhages and diffuse axonal injuries [33]. Fast protocols allow scanning without sedation in many children, although most supporting evidence comes from clinically stable patients [34]. fast MRI was directly compared to CT in children with head trauma and showed high feasibility and diagnostic agreement, supporting its potential role as a radiation-free alternative in selected ED populations [35]. Despite this, limited access, longer acquisition times, and logistical constraints restrict its routine use in emergency care. POCUS is increasingly used to detect skull fractures in children. When performed by trained emergency clinicians, POCUS has demonstrated high accuracy, with a sensitivity of 91% and specificity of 96%, confirming its effectiveness in skull fracture detection [32]. However, CT is not required for all skull fractures, particularly linear, non-displaced ones in well-appearing, asymptomatic children. There is concern that increased use of POCUS could unintentionally drive CT use by identifying non-clinically significant fractures. Current evidence does not yet establish a clear link between ultrasound findings and risk of clinically important TBI (ciTBI), and more data are needed before POCUS can be broadly recommended as a decision tool for CT selection. Importantly, certain age groups remain underrepresented in decision rule validation studies. Cranial ultrasound (CUS) performed by trained radiologists has shown high diagnostic accuracy in infants with minor head trauma. A retrospective study involving over 300 infants reported a sensitivity of 93% and specificity of 98% for detecting skull fractures and intracranial haemorrhage [36]. Whilst this approach is not universally available and differs from emergency physician-performed POCUS, it may provide a valuable, radiation-free alternative in centres with radiology expertise. NIRS uses infrared light absorption patterns to identify intracranial haematomas by comparing optical densities at specific points on each side of the cranium. It has shown potential for the identification of intracranial haematomas in adults, but there have been few paediatric trials [37]. Whilst NIRS has shown some promise in adult trauma care, paediatric data remain limited. In one study, it was well-tolerated by children, with scan times averaging under six minutes [38]. However, the technology is currently unreliable in injuries over 12 h old, cannot detect very small or deep haematomas, and can be confounded by scalp or bilateral intracranial haematomas [19]. Blood-based biomarkers are an area of rapidly evolving research in paediatric TBI. A systematic review identified 21 studies examining 14 different biomarkers [39]. The most extensively studied were S100B, ubiquitin carboxy-terminal hydrolase L1 (UCH-L1) and glial fibrillary acidic protein (GFAP). A meta-analysis has shown promising results for the use of S100B as part of a rule-out strategy, with a pooled sensitivity of 100% (but a specificity of 34%) for positive CT findings [40]. Whilst these results are promising, there is significant heterogeneity in study design, thresholds, and outcome definitions. The [20] guidelines concluded that these biomarkers are not yet suitable for routine clinical use in acute mild TBI due to insufficient evidence [20]. Whilst biomarker research continues to advance, these tools are not currently recommended for routine use in emergency settings and remain investigational.

In summary, whilst these tools offer significant future promise, particularly in reducing radiation exposure or identifying subclinical injuries, none are currently recommended for routine decision-making in mild TBI in the emergency setting. Clinical assessment, validated decision rules, and selective CT use remain the gold standard.

## Approach to moderate and severe TBI: priorities in the ED

Whilst the management of moderate and severe TBI is complex and extensively detailed in guidelines such as those from the Traumatic Brain Injury Foundation [41], a comprehensive discussion is beyond the scope of this review. Instead, this section highlights core principles relevant to early ED care. The primary goal in the ED is to minimise secondary brain injury by ensuring adequate oxygenation, perfusion, and physiological stability. A structured primary survey (ABCDE) should be performed immediately, with early intubation and ventilation often required in severe TBI, and sometimes in moderate TBI to facilitate neuroimaging or protect the airway [42]. The child should be positioned with the head elevated to 30 degrees and the neck in midline, avoiding any venous outflow obstruction; tight tube ties and cervical collars should be removed if safely possible [43]. A systematic primary survey should be performed, and any life-threatening conditions should be managed as they are found. Intubation and ventilation are usually required in severe TBI for airway protection, but they may also be needed in moderate TBI to allow for neuroimaging and the instigation of basic neuroprotective measures. Key targets include avoiding hypoxia and hypotension, maintaining normoglycaemia and normothermia [42], and restoring normovolaemia with isotonic fluids or blood products as appropriate. Hypotension should be avoided; if fluid resuscitation is required, isotonic fluids or blood should be administered (depending on the source of fluid loss) with the aim of restoring normovolaemia. Most guidelines recommend cardiovascular targets based on cerebral perfusion pressure rather than blood pressure, which is often a challenge for ED clinicians, as intracranial pressure (ICP) monitoring is rarely established in the department. If there is a suspected rise in intracranial pressure (ICP), basic measures are insufficient; stabilisation should include sedation, analgesia, seizure control, hyperosmolar therapy (e.g. mannitol or hypertonic saline), and short-term hyperventilation only if signs of herniation are present [42]. Tranexamic acid (TXA) is also recommended following the CRASH-3 trial, with [20] guidelines advising a 15–30 mg/kg (maximum 2 g) IV bolus for children with severe TBI and no active extracranial bleeding [20] [44]. Rapid recognition, early stabilisation, and prompt transfer to definitive care are essential, and management should follow locally agreed trauma and neurosurgical pathways to avoid delays in critical interventions.

## Prognosis of severe TBI and strategies for improving outcomes

Mortality rates in severe TBI are based predominantly on small retrospective cohort studies. Estimates range between 15 and 35%, and of those who survive, between 11 and 61% have medium or severe disability [45]. Mortality relates to primary injury factors such as injury severity (including extracranial injuries) and initial GCS, and to secondary factors such as hypotension, hypoxia, coagulopathy, and hyperglycaemia [45]. ED management should therefore aim to minimise secondary injury by optimising physiology as early as possible. Early attention to details such as thromboprophylaxis and skin integrity is also important to prevent later complications that contribute to morbidity.

## Prognosis of mild TBI in children: advice for patients and families

The true incidence of persistent post-concussive symptoms (PPCS) remains largely unknown. A large prospective cohort study from Canada reported rates of 31% at 28 days post-injury. This study derived and validated a clinical risk score for predicting persistent symptoms at 28 days for use in the acute setting. The nine-point score encompasses demographic data, injury history, initial symptoms, physical examination, and cognitive function to stratify patients into low, medium, and high risk of persistent symptoms [46]. Additionally, another study conducted in a paediatric ED also examined the use of a similar clinical risk score to predict PPCS. It found that factors such as headaches and a history of prolonged concussion recovery were strong predictors of PPCS. This study supported the score’s utility for risk stratification, further reinforcing its practical application in clinical settings. Whilst the original score may still require validation in broader contexts, the evidence from this second study provides robust support for its effectiveness in EDs, suggesting it may already be a valuable tool for guiding patient care [47]. Beyond PPCS, population-based research has shown an association between paediatric TBI and an increased risk of developing psychotic syndromes later in life. A recent nationwide study found the highest risk during the first 10 years post-injury, with female patients particularly affected [48]. These findings highlight the need to consider both short- and long-term neuropsychiatric outcomes when counselling families and planning follow-up.

## Future directions in paediatric TBI research

Future research in paediatric TBI should focus on improving early diagnosis, personalising treatment, and enhancing long-term outcomes across the spectrum of injury severity. Refinement of diagnostic tools such as validated biomarkers and advanced imaging techniques like fast MRI may allow earlier and more accurate identification of ciTBI whilst reducing unnecessary CT use and radiation exposure. There is a significant gap in evidence for pharmacological interventions in paediatric mild TBI. Future work should prioritise the evaluation of medications that may prevent or reduce prolonged symptomatology, as current treatment strategies are almost entirely supportive and non-specific. Longitudinal studies are also needed to better understand the long-term effects of mild TBI, particularly in relation to persistent symptoms and post-concussion syndrome. Recovery modifiers such as age, biological sex, injury history, and psychosocial stressors should be examined to support more individualised care planning. In moderate to severe TBI, further research should focus on developing age-appropriate neuroprotective therapies, improving post-acute rehabilitation models, and reducing the risk of long-term disability. Multimodal strategies addressing physical, cognitive, and emotional recovery remain critical for improving outcomes. Priority areas for future research include validating clinical decision rules in preverbal children, studying TBI outcomes in neurodivergent populations, and assessing the feasibility of implementing MRI-based diagnostic protocols in real-world ED workflows.

Finally, future work should support evidence-based approaches to school reintegration and long-term recovery for children with persistent symptoms. Involving families, schools, and community services in individualised recovery plans will be key to improving functional outcomes and quality of life. It is also essential that future research and guideline development consider disparities in access to imaging and decision-support tools across health systems. Adaptable, resource-sensitive strategies are needed to ensure equitable care in low-resource environments.

## Key messages for emergency clinicians

- Most paediatric TBIs are mild; use decision rules (e.g. PECARN) to guide imaging.
- Observation is safe for low-risk cases; CT is not always necessary.
- Biomarkers and advanced imaging are not ready for routine ED use.
- In severe TBI, focus on stabilisation and preventing secondary injury.
- Imaging access and guideline implementation vary globally; care must be adapted to local context.

## Data Availability

No datasets were generated or analysed during the current study.
